# Stabilizing Exposure of Conserved Epitopes by Structure Guided Insertion of Disulfide Bond in HIV-1 Envelope Glycoprotein

**DOI:** 10.1371/journal.pone.0076139

**Published:** 2013-10-16

**Authors:** Aemro Kassa, Antu K. Dey, Pampi Sarkar, Celia Labranche, Eden P. Go, Daniel F. Clark, Yide Sun, Avishek Nandi, Karin Hartog, Heather Desaire, David Montefiori, Andrea Carfi, Indresh K. Srivastava, Susan W. Barnett

**Affiliations:** 1 Vaccines Research, Novartis Vaccines & Diagnostics Inc., Cambridge, Massachusetts, United States of America; 2 Vaccines Research, Novartis Vaccines & Diagnostics Inc., Emeryville, California, United States of America; 3 Department of Surgery, Duke University Medical Center, Durham, North Carolina, United States of America; 4 Department of Chemistry, University of Kansas, Lawrence, Kansas, United States of America; University of Massachusetts Medical Center, United States of America

## Abstract

Entry of HIV-1 into target cells requires binding of the viral envelope glycoprotein (Env) to cellular receptors and subsequent conformational changes that culminates in fusion of viral and target cell membranes. Recent structural information has revealed that these conformational transitions are regulated by three conserved but potentially flexible layers stacked between the receptor-binding domain (gp120) and the fusion arm (gp41) of Env. We hypothesized that artificial insertion of a covalent bond will ‘snap’ Env into a conformation that is less mobile and stably expose conserved sites. Therefore, we analyzed the interface between these gp120 layers (layers 1, 2 and 3) and identified residues that may form disulfide bonds when substituted with cysteines. We subsequently probed the structures of the resultant mutant gp120 proteins by assaying their binding to a variety of ligands using Surface Plasmon Resonance (SPR) assay. We found that a single disulfide bond strategically inserted between the highly conserved layers 1 and 2 (C65-C115) is able to ‘lock’ gp120 in a CD4 receptor bound conformation (in the absence of CD4), as indicated by the lower dissociation constant (Kd) for the CD4-induced (CD4i) epitope binding 17b antibody. When disulfide-stabilized monomeric (gp120) and trimeric (gp140) Envs were used to immunize rabbits, they were found to elicit a higher proportion of antibodies directed against both CD4i and CD4 binding site epitopes than the wild-type proteins. These results demonstrate that structure-guided stabilization of inter-layer interactions within HIV-1 Env can be used to expose conserved epitopes and potentially overcome the sequence diversity of these molecules.

## Introduction

Approximately 34 million people are infected with Human Immunodeficiency Virus type 1 (HIV-1) [1]. Although the epidemic may have stabilized to some extent in many regions including parts of sub-Saharan Africa [2], where the burden of the disease has been most severe, a vaccine against HIV-1 remains a huge unmet medical need globally. Although HIV-1 infected subjects are able to mount a humoral immune response directed against the viral envelope glycoprotein (Env) during initial HIV-1 infection, the Env-specific antibodies generated in large part fail to neutralize the virus nor impact disease progression [3]. This failure is due to multiple mechanisms of immune evasion inherent to HIV Env that protect the virus against the host's antibody responses. These include a high degree of sequence variability in the viral Env antigen primarily in its immunodominant “variable” loops, carbohydrate masking of underlying conserved polypeptide epitopes, occlusion and conformational masking of key conserved epitopes and molecular mimicry of self-antigens [4]. Nevertheless, despite the large degree of Env variability, the virus must maintain certain conserved sites that bind cellular receptors to mediate cell entry. These conserved receptor binding sites in HIV-1 gp120, which include the CD4-binding site (CD4BS) and CD4-induced (CD4i; co-receptor) binding sites, are potentially vulnerable targets for antibody-mediated neutralization.

**Figure 1:**
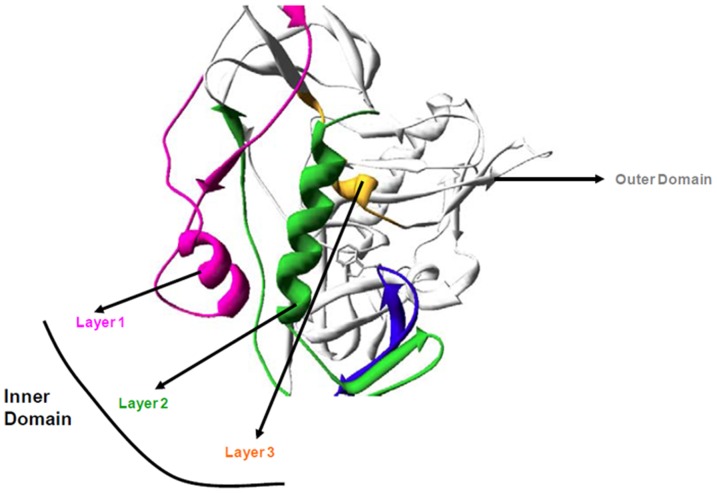
Schematic representation of layered organization of the gp120 inner domain. Ribbon diagram representation showing the inner domain of gp120 and its organization into three layers: layer 1 (magenta), 2 (green) and 3 (orange). The outer domain of gp120 is shown in white/gray. The representation is based on RCSB PDB ID - 3JWO [6].

Recent high-resolution gp120 structures [5], [6] have revealed the complete structure of the inner domain of HIV-1 gp120 that was missing from previous studies [7]–[10]. These structures showed that the inner domain of gp120 in CD4-bound state is organized into 3 layers (layers 1, 2 and 3), projecting from a β-sandwich structure towards the target cell membrane (Figure 1) [5], [6], [11], [12]. This domain was identified as the primary region of gp120 that undergoes extensive conformational changes as it transitions from the unliganded to CD4-bound state [5], [13], [14]. Subsequent to CD4 binding, conformational changes in the inner domain contribute to two essential downstream events required for entry of the virus into target cells. These include: (i) stabilization of the initial weak gp120-CD4 contacts, and (ii) exposure of a conserved site on gp120 for co-receptor binding. In the CD4-bound state of gp120, the three layers of the inner domain (Figure 1) come together through specific interlayer interactions and stabilize the CD4-bound conformation of the viral envelope glycoprotein.

The interactions between layers 1 and 2 are mediated through a number of residues identified by site-directed mutagenesis [11], [12]. Disruption of these inter-layer interactions led to destabilization of the CD4-bound conformation of gp120 resulting in reduced affinity to CD4 [11]. Similarly, mutations that reduced interactions between the layers also prevented binding of gp120 to small molecule CD4-mimetics, such as NBD-556, which require CD4-bound conformation of the gp120 for a high affinity interaction [11], [15]. Binding of antibodies, such as 17b, 48d and 412d that recognize the CD4-induced conserved binding sites on gp120 were also dramatically reduced by mutations that prevented inter-layer interaction in the inner domain [11]. In general, reduced inter-layer interactions led to reduced exposure of the conserved binding sites (i.e. CD4 and CD4-induced/co-receptor binding sites) and therefore reduced binding of ligands recognizing these sites.

Previous structure-guided attempts to stabilize gp120 in a receptor-bound conformation utilized various approaches including a combination of disulfide bridges and cavity-filling mutations [16]–[18]. The largest degree of stabilization was achieved by the formation of disulfide bridges after targeted substitution with cysteine residues [16]–[18]. While these disulfide-stabilized structures achieved exposure of the CD4-induced binding sites in gp120, they also reduced exposure of the CD4-binding sites, as measured by affinity of CD4 & CD4-binding site antibodies. This was probably due to direct interference of some of the newly inserted disulfide bridges with antibody binding. In addition, most of previously tested disulfide-inserting mutations focused on stabilizing the inner and outer domain contacts [16]–[18], rather than restricting movement within the inner domain. However, as shown in recent work and described above, the inter-layer interactions within the inner domain are crucial in determining the transition of gp120 to CD4-bound conformation [5], [6], [11], [12]. Therefore, unlike previous attempts, we have focused here in stabilizing the inter-layer contacts within the inner domain by inserting a disulfide linkage far from the receptor binding area. We hypothesized that stabilization of the interlayer contacts would put gp120 into a conformation in which both the CD4 and CD4-induced binding sites are exposed.

We, therefore, mutated residues at strategically selected sites in layers 1 and 2 (and layer 3) into cysteines to allow formation of disulfide bridges that would stabilize the inner domain of gp120. Analysis of binding affinities of CD4 and anti-gp120 monoclonal antibodies (mAb) to wild-type gp120 and the final selected stabilized gp120 (gp120 L1-SS-L2) revealed that the stabilized protein not only recognized soluble CD4 (sCD4) and CD4BS-antibodies with similar affinity as wild-type but also recognized CD4-induced antibody, 17b, with much higher affinity than wild type and in the absence of sCD4. These stabilized recombinant Env glycoproteins, both the monomeric gp120 and trimeric gp140, were shown to elicit robust high titer and high avidity antibody responses in rabbits, with the stabilized antigens generating more “balanced” responses directed to multiple gp120 epitopes with a higher proportion of antibodies directed against both the CD4i and CD4 binding site epitopes. These results demonstrate that structure-guided stabilization of inter-layer interactions within HIV-1 Env can be used to expose conserved epitopes and potentially overcome the structural diversity of these molecules when used as vaccine immunogens.

## Results

### Structural analysis of proximal residues between layers allows introduction of appropriate cysteine pairs for generation of disulfide-stabilized gp120 and gp140 proteins

We examined all residues that lie at the interface of layers 1, 2 and 3, and attempted to identify residues that could be targeted for substitution with cysteine for disulfide bond formation. In order for a disulfide bridge to be formed between two cysteine residues, the side chains have to be at the proper distance and in the right orientations with respect to each other. We, therefore, used the distance between C-β (Carbon–beta) atoms as one of the criteria for selecting residues that would be substituted with cysteines. The average distances between C-β atoms of cysteines that formed disulfide bridges in previously determined gp120 structures was in a range of 3.5Å – 4.7Å [6], [17]. In addition, we also examined a crystal structure of gp120 into which specific amino acids had been mutated in disulfide bond forming cysteines, W96C-V275C and I109C-Q428C) [17], and observed that the C-β distances of the native and cysteine substituted residues, which formed disulfide bonds, were 4.1Å and 4.3Å, respectively. Therefore, we screened and selected for residues on interacting layers that had C-β-C-β distances falling within this (3.5Å – 4.7Å) range.

Based on the C-β distance criteria and expression analysis of a subset of cysteine mutants that were designed *in silico* and tested for expression and antigenicity (binding to CD4 and CD4i-directed antibody, 17b) post-purification, we identified a pair of residues that could be substituted with cysteine for disulfide bridging of layers. Except for one, all mutations in gp120 either failed to express or could not be purified as homogenous monomeric, disulfide-stabilized gp120 that was functionally active (i.e., bound CD4 and underwent CD4-induced conformational change to allow CD4-directed antibody, 17b, binding) (Table S1). Therefore, the targets chosen for cysteine substitution to bridge layers 1 and 2 were Valine 65 (from layer 1) and Serine 115 (from layer 2), (Figure S1A & B). The C-β atomic distance between these residues in the new CD4 bound gp120 structure is 3.7Å (Figure S1B, [6]), which was a sufficient distance for disulfide bridging.

**Figure 2:**
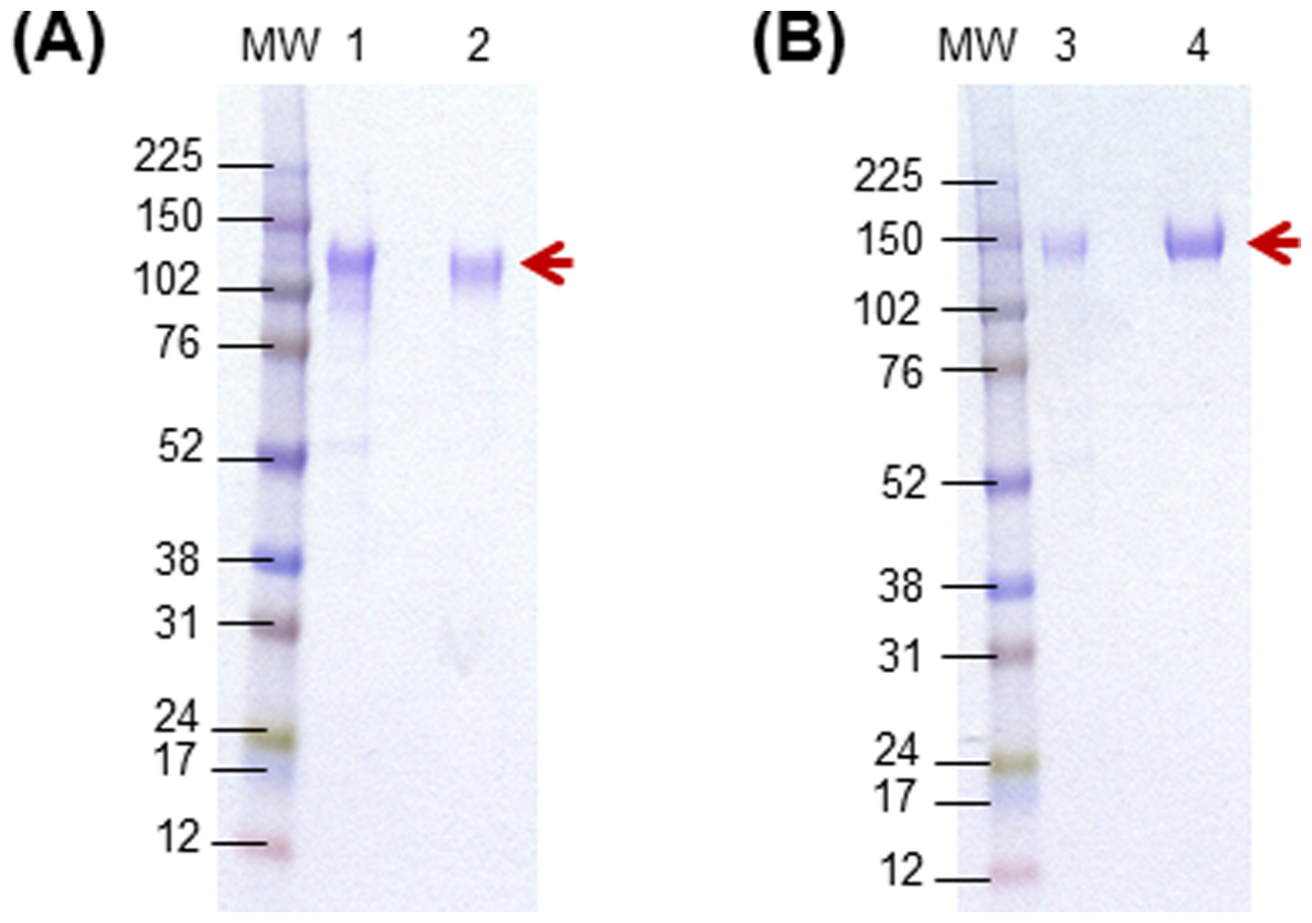
Expression and purification of disulfide-stabilized SF162 gp120 and gp140. The two disulfide-stabilized proteins are referred to as gp120 L1-SS-L2 and gp140 L1-SS-L2. The SS refers to the disulfide bond. SDS-PAGE followed by coomassie-staining to show purity of GNA-lectin affinity column and DEAE-column purified (A) Disulfide stabilized SF162 gp120 L1-SS-L2 (lane 1) and wild-type gp120 (lane 2), and (B) Disulfide stabilized SF162 gp140 L1-SS-L2 (lane 3) and wild-type gp140 (lane 4). MW refers to Molecular Weight standard/marker. The gp120 s and gp140 s are indicated by the red arrow.

These two residues, Valine 65 and Serine 115, were mutated to cysteine in both gp120 and a cleavage-defective gp140 [19], [20] backbone from HIV-1_SF162_. Both recombinant disulfide-stabilized gp120 and cleavage-defective gp140 proteins, along with their respective wild-type forms, were then expressed via transient transfection of HEK 293 cells and purified using an established purification protocol ([19], and Materials and Methods). All four wild-type and disulfide-stabilized proteins were purified to >95% purity, as visualized using a coomassie-stained SDS-PAGE (Figure 2). The disulfide-stabilized gp120 and gp140 are referred to as gp120 L1-SS-L2 (L1 – layer 1; SS – disulfide linkage; L2 – layer 2) and gp140 L1-SS-L2, respectively.

To ascertain that disulfide bond was formed in gp120 via the engineered cysteine residues at position 65 and 115, we used mass spectrometry. The LC/ESI-FTICR MS data confirmed that disulfide bond between engineered cysteines at position 65 and 115 was formed and detected in major forms of disulfide-linked tryptic peptides (Figures S2 and S4).

### Insertion of a disulfide linkage increases ligand affinity to CD4-induced site without affecting CD4 receptor binding

**Figure 3:**
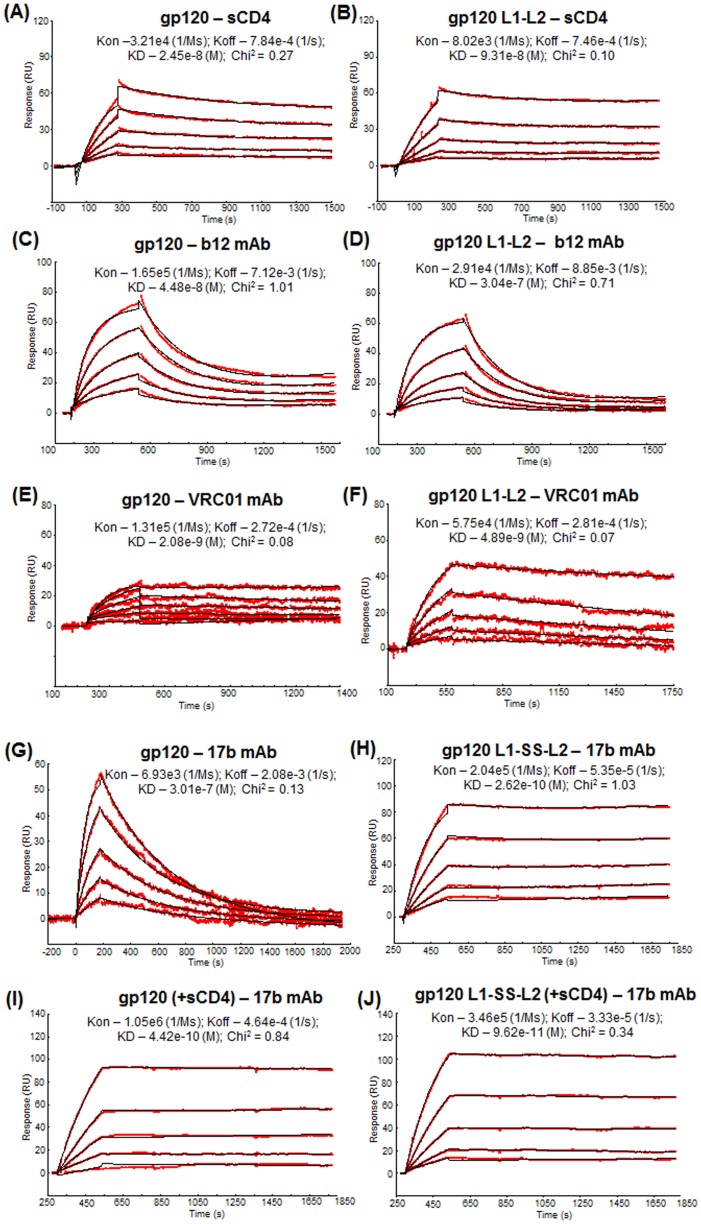
Binding affinity analysis of binding of wild-type and disulfide-stabilized SF162 gp120 to sCD4 and mAbs b12 and 17b. Overlay of binding of varying concentrations of wild-type SF162 gp120 to (A) sCD4, (C) b12 mAb, (E) VRC01 mAb, (G) 17b mAb and (I) 17b mAb (+sCD4). Overlay of binding of varying concentrations of disulfide-stabilized SF162 gp120 (gp120 L1-SS-L2) to (B) sCD4, (D) b12 mAb, (F) VRC01 mAb, (H) 17b mAb and (J) 17b mAb (+sCD4). ∼250–500 RUs of sCD4 or mAbs, b12, VRC01 or 17b, were immobilized directly onto a CM5 sensor chip via amine coupling. Varying concentrations of gp120s, either wild-type of disulfide-stabilized, were then injected at 80 µl/min, at 25°C with HBS-EP buffer as running buffer. The experimental curves were then fitted to a 1∶1 Langmuir binding model using BIAevaluation software 3.2 (BIAcore Inc). The red lines indicate the experimentally derived curves while the black lines indicate curves generated after fitting the experimental data to the 1∶1 Langmuir binding model (BIAevaluation software 3.0). Kon (association rate, in 1/Ms), Koff (dissociation rate, in 1/s) and Kd (dissociation constant, in M) are indicated for each paired (ligand-analyte) interaction, derived by fitting the experimental data to the 1∶1 Langmuir binding model. Chi2 (of ≤1) refers to goodness of fit of the statistical model to observed experimental data.

Following structural “fixation” of gp120 and gp140 by the inserted disulfide bond, we utilized Surface Plasmon Resonance (SPR) to evaluate the differences in binding affinities between wild-type and disulfide-stabilized (L1-SS-L2) gp120 proteins to a CCR5 peptide (Figure S3) and soluble CD4, as well as mAbs VRC01, b12, and 17b (−/+ sCD4) (Figure 3), to determine if the molecules were indeed ‘locked’ into a CD4 bound state and to what degree the conserved receptor and co-receptor binding sites were exposed.

Initial qualitative binding of wild-type and disulfide-stabilized gp120 proteins, in absence or presence of sCD4, to a 22-mer tyrosine-sulfated biotinylated N-terminal CCR5 peptide [21] confirmed the improved exposure of the CD4-induced co-receptor binding site in the disulfide-stabilized gp120 L1-SS-L2 protein (Figure S3). Following this, we carried out an in-depth binding affinity analysis of the two gp120 proteins to CD4, VRC01, b12, and 17b (−/+ sCD4). When the binding of wild-type and disulfide-stabilized gp120 proteins to soluble CD4 (sCD4) was analyzed, we observed that the stabilized gp120 protein bound to CD4 with >3.5-fold lower affinity than the wild-type gp120 (gp120 wild-type-sCD4: 24.5 nM; gp120 L1-SS-L2-sCD4: 93.1 nM) (Figure 3A & B). This difference in affinity is primarily attributed to the difference in on-rate (*k_on_*) while the off-rates (*k*_off_) are near-identical (Figure 3). Since binding of gp120 to CD4 requires elements of gp120's inner domain, outer domain and the bridging sheet, the presence of the disulfide bridge in the inner domain likely restricted the inherent flexibility of the molecule thereby affecting the accessibility needed for initial “docking” of CD4 to gp120, and hence the lower affinity of the stabilized gp120 for CD4 [17]. The affinity between gp120 (wild-type or other forms) and CD4 (4-domain, 2-domain or CD4-IgG2), measured previously particularly via non-SPR based methods [22], [23], showed sub-nanomolar affinity. The observed relative difference in affinity via non-SPR methods could be due to differences in the strains of the gp120 used, the expression system and level of purity of the gp120 protein, the buffer systems used and/or their linear range of quantitation. More importantly, in ELISA the measured affinity is not based on 1∶1 binding and hence can be stronger than the 1∶1-derived affinity from SPR presented here. When binding to CD4BS-mAb b12 was compared, we observed a similar trend but a greater fold-difference. While wild-type gp120 bound with an affinity of 44.8 nM, the disulfide-stabilized gp120 bound with ∼7-fold lower affinity (Kd – 304 nM) (Figure 3C & D). Binding to more potent CD4BS-mAb VRC01 showed a similar binding trend in that the disulfide-stabilized gp120 bound VRC01 mAb with (∼2-fold) lower affinity, predominantly attributed to the on-rate (*k_on_*) (Figure 3E & F).

When we compared binding to various mAbs, such as 17b, which binds to the CD4-induced (CD4i) epitope(s), we found that while unliganded, wild-type gp120 bound to 17b with an affinity of 301 nM, the unliganded, stabilized gp120 bound with ∼700-fold higher affinity (Kd – 0.44 nM) (Figure 3G & H). Upon ligation with CD4 (gp120+sCD4), the wild-type gp120 now bound mAb 17b with significantly higher affinity (Kd – 0.26 nM), which was only ∼2-fold better than the affinity with unliganded, stabilized gp120. The sCD4-liganded, stabilized gp120 (gp120 L1-SS-L2 + sCD4), on the other hand, bound 17b with ∼5-fold higher affinity (Kd - 0.096 nM) than unliganded, stabilized gp120 (gp120 L1-SS-L2) (Kd – 0.44 nM) and ∼3-fold higher affinity than sCD4-liganded, wild-type gp120 (gp120+sCD4) (Kd – 0.26 nM) (Figure 3I & J). Overall, when comparing the affinities to mAb 17b that is dependent on CD4-binding, we observed that the disulfide stabilization was able to induce a similar level of exposure to what sCD4 binding induced in a wild-type protein. In addition, we observed that when sCD4 was pre-incubated to the disulfide-stabilized gp120 (gp120 L1-SS-L2+sCD4), a further (∼5-fold) increase in affinity (driven predominantly by increased on-rate) was observed, indicating that the stabilized gp120 was not fully ‘locked’ and/or CD4 can induce a further conformational alteration and stabilization to enable optimal 17b-binding. These data confirmed the stabilization of the inter-layer contacts of inner domain enhanced exposure of CD4-induced epitopes on gp120 in the absence of the CD4 receptor.

### Disulfide-stabilized gp120 and gp140 elicited high titer and high avidity antibody responses in rabbits

The *in vitro* binding analysis of the gp120 antigen indicated that the disulfide-stabilized molecules were likely ‘locked’ into the intended CD4-bound conformation while preserving exposure of the CD4 binding site. We, therefore, grafted those mutations onto the gp140 backbone and generated a similarly disulfide-stabilized protein, gp140 L1-SS-L2, so that we could evaluate both monomeric (gp120) and trimeric (gp140) forms of the antigen. Due to an inability to fit the gp140-sCD4/mAb SPR binding analysis to the 1∶1 Langmuir binding model, we were unable to report a similar degree of SPR analysis on the stabilized-gp140 protein. Regardless, we wanted to compare the immunogenicity of the two disulfide-stabilized proteins, gp120 L1-SS-L2 and gp140 L1-SS-L2, to their wild-type counterparts.

**Figure 4:**
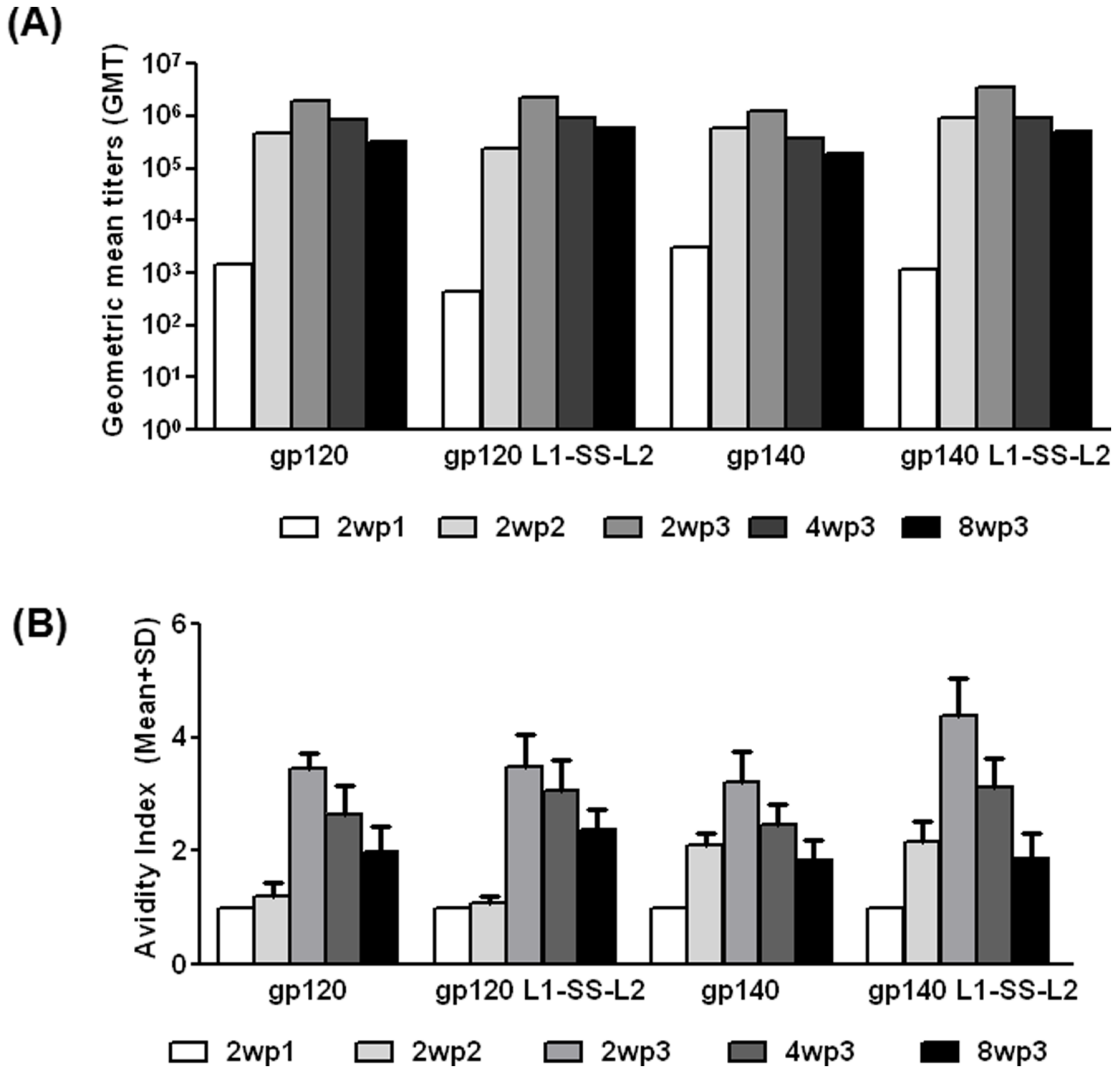
Binding analysis of serum antibodies post-immunization. Env binding analysis of serum antibodies from an immunogenicity study in rabbits using a gp120/gp140 protein-only regimen with Carbopol971P + MF59 [54] as adjuvant. (A) Geometric mean (binding) titers and (B) Avidity of anti-Env antibodies in sera collected before the onset of immunization (pre-bleed) and at 2wp2, 2wp3, 4wp3 and 8wp3 (wp - weeks post) immunization time-points.

Four groups of 5 rabbits each were immunized at 0, 4, and 12 weeks with 25 µg of well-characterized preparations of each Env proteins using a stable vaccine formulation of the potent Carbopol 971P + MF59 as adjuvant [24]. Env-specific binding antibody titers and avidities were evaluated in sera following each immunization. We observed that while the 1^st^ immunization generated 10^3^−5×10^3^ geometric mean titers (GMT), the 2^nd^ protein immunizations improved the titers by ≤3−logs (5×10^5^−10^6^); however, after the 2^nd^ immunization, the GMTs for all groups were comparable and only an incremental improvement, to a maximal of ≥10^6^−5×10^6^ GMTs, was observed after the 3^rd^ immunization. (Figure 4A). The GMTs declined at 4wp3, and further at 8wp3 time-points and the rate of decay was similar in all groups.

When we measured the avidities of the anti-Env antibodies generated, we observed that after the 1^st^ immunization, all groups elicited similar levels of low avidity antibodies (Figure 4B). After the 2^nd^ immunization, in the gp120 groups, there was little or no increase in avidities; in contrast, the gp140-immunized groups (both wild-type and disulfide-stabilized) showed a ≥2-fold increase. The 3^rd^ immunization resulted in increasing avidities further, a remarkable ≥4-fold in the gp120-immunized groups and 1.5- (gp140) to 2.5- (gp140 L1-SS-L2) fold increased avidity in gp140-immunized groups (Figure 4B). At 2wp3, the disulfide-stabilized gp140 generated the highest avidities in comparison to all other groups. When we followed the antibody avidity after the end of the 3^rd^ and final immunization, we observed a steady but comparable decrease across all groups in avidities at 4wp3 and 8wp3 time-points - a trend similar to that seen with antibody GMT titers (Figure 4A).

**Figure 5:**
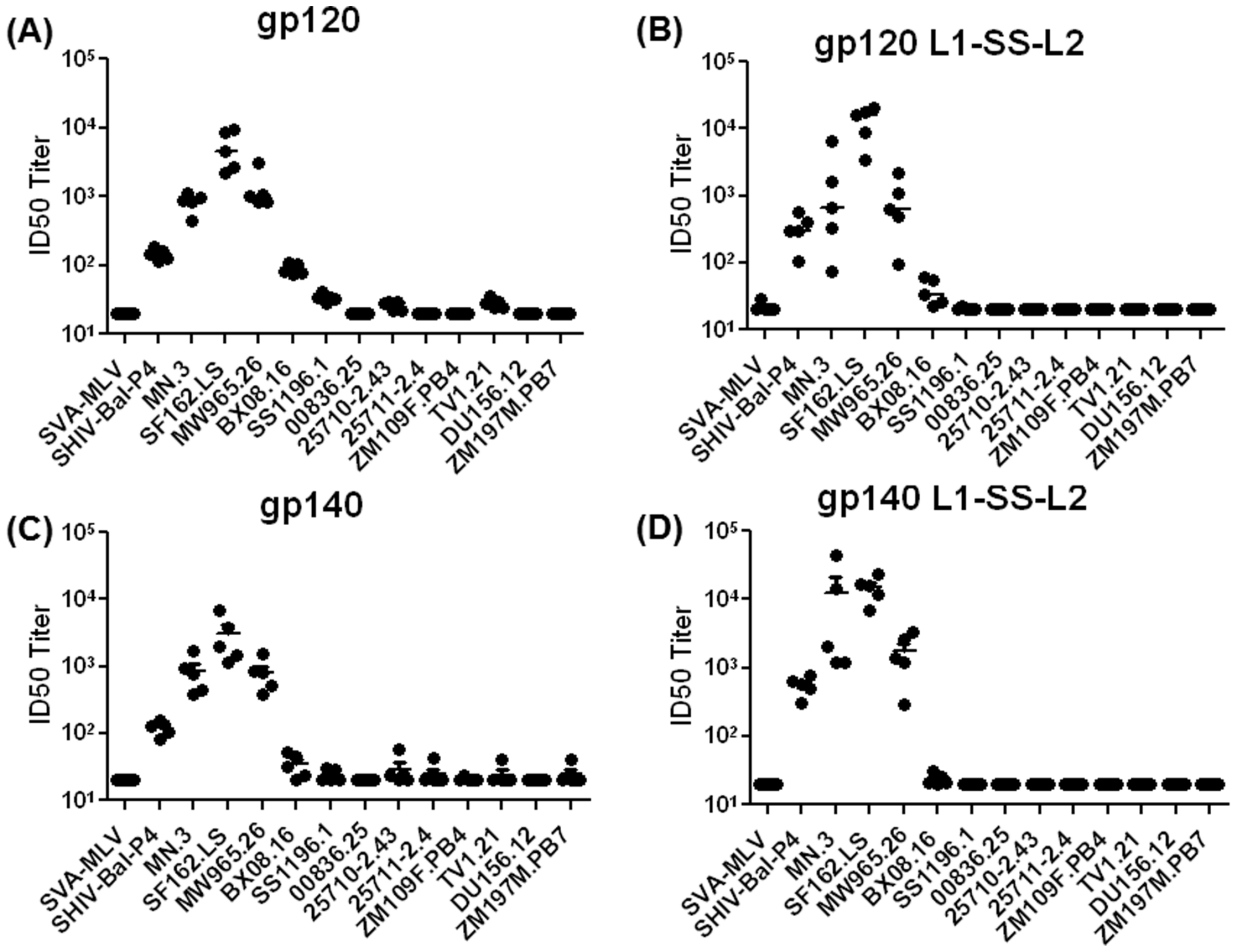
Neutralization of pseudoviruses using TZM-bl luciferase-based assay. Neutralization of control (SVA-MLV), SHIV-chimeric (SHIV-Bal-P4) and HIV-1 pseudoviruses encompassing Tier-1A/B [MN.3 (Subtype B), SF162.LS (Subtype B), MW965.26 (Subtype C), Bx08.16 (Subtype B), SS1196.1 (Subtype B), 00836.25 (Subtype C), 25710-2.43 (Subtype C), 25711-2.4 (Subtype C), ZM109F.PB4 (Subtype C), TV1.21 (Subtype C), Du156.12 (Subtype C), ZM197M.PB7 (Subtype C)] isolates by 2wp3-sera from rabbits immunized with (A) gp120, (B) gp120 L1-SS-L2, (C) gp140, or (D) gp140 L1-SS-L2.

When we tested the serum antibodies from the 2wp3 time-point (peak titers and avidities) for virus neutralization against a panel of Tier 1 pseudoviruses, we observed that all immunogens were able to raise immune responses that neutralized only 4–5 out of 13 pseudoviruses. Those pseudoviruses included SHIV-Bal-P4 (subtype B), MN.3 (subtype B), SF162.LS (subtype B), MW965.26 (Subtype C) and BX08.16 (subtype B). Among the animals immunized with gp120 immunogens, the only significant difference was that the disulfide-stabilized gp120 generated approximately log-higher antibodies against the homologous (SF162.LS) virus; the ID50 titers against the rest 4 heterologous viruses were comparable. Among the animal immunized with gp140 immunogens, the disulfide-stabilized gp140 generated a log-higher antibodies against all pseudoviruses (SHIV-Bal.P4, MN.3 and SF162.LS), except MW965.26 where the ID50 titers were comparable (Figure 5, A-D).

### Disulfide-stabilized gp140 generated a more balanced epitope-specific binding antibody response

From the binding and neutralizing antibody analyses (Figure 4 and 5), we observed that the disulfide-stabilized gp140 generated higher avidity and slightly improved neutralizing antibody responses to Tier 1 pseudovirions at the 2wp3 time-point. We therefore used the sera from the 2wp3 time-point to map the epitope-specificity of the Env-specific antibodies.

**Figure 6:**
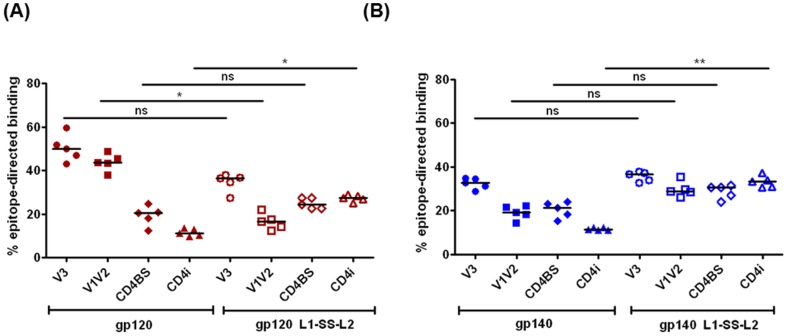
Dissection of epitope-specific recognition of antibodies from vaccine sera. Analysis of 2wp3-sera from rabbits for dissecting specificity of epitopes recognized (expressed as ‘% epitope-directed binding’) by the antibodies elicited when immunized with (A) wild-type gp120 (red solid symbols) or gp120 L1-SS-L2 (red open symbols) or (B) wild-type gp140 (blue solid symbols) or gp140 L1-SS-L2 (blue open symbols). The percent epitope-directed response is calculated as follows: EC50(mutant)/EC50(wild-type)x100. ns – non-significant (p>0.05); * <0.05; ** <0.001.

To do so, we generated four SF162 gp120 mutants: gp120ΔV3 (to map reactivity to V3 loop), gp120ΔV1V2 (to map reactivity to V1V2 loop), gp120D368R (to map reactivity to CD4-binding site, CD4BS) [25]–[28] and gp120I420R (to map reactivity to CD4-induced, CD4i, site) [26]–[29]. These proteins were transiently expressed in HEK 293 cells and purified using the same protocol described for the gp120/gp140 immunogens. The gp120 mutants were evaluated by testing their reactivity (or lack thereof) to CD4-IgG2 and epitope-directed mAbs (data not shown) [28]. Following this confirmation, the gp120 mutants were then used in ELISA to evaluate the 2wp3-sera for reactivity to the four epitopes in question – V3, V1V2, CD4BS and CD4i-site. We observed that 2wp3-sera from gp120-immunized rabbits elicited ∼45–60% of total binding antibodies directed to the V3-loop and 40–50% of antibodies to the V1V2-loop, and a much lower fraction to the CD4BS (15–25%) and the CD4i-site (10–14%) (Figure 6A, filled red symbols). When the rabbits were immunized with the disulfide-stabilized gp120 (gp120 L1-SS-L2), we observed differences in overall percent reactivity of the Env-specific antibodies: ≤30–35% anti-V3 antibodies, ≤15–20% anti-V1V2 antibodies, ≥22–27% anti-CD4BS antibodies and ∼25–30% anti-CD4i-site antibodies, with less fractional Ab reactivity to the variable loops and more to the CD4BS and CD4i sites (Figure 6A, open red symbols). In rabbits immunized with gp140 protein, we observed similar trend for epitope-specificity as that of gp120 L1-SS-L2, except for ∼3-fold reduced CD4i-antibodies: ≤30–35% anti-V3 antibodies, 18–22% anti-V1V2 antibodies, ∼15–23% anti-CD4BS antibodies and 11–12% anti-CD4i-site antibodies (Figure 6B, blue filled symbols). However, when we used the disulfide-stabilized gp140 (gp140 L1-SS-L2) as immunogens, all rabbits in this groups showed similar levels (≥25–35%) of specificity to all four epitopes: ∼32–36% anti-V3 antibodies, 25–34% anti-V1V2 antibodies, ∼25–30% anti-CD4BS antibodies and >30–35% anti-CD4i-site antibodies (Figure 6B, blue open symbols). Overall, the disulfide-stabilized gp140 (gp140 L1-SS-L2) generated the most “balanced” epitope-specific response with similar titers of Abs recognizing each of the specificities evaluated, in comparison to the wild-type gp120, disulfide-stabilized gp120 and wild-type gp140 immunogens. Unlike the gp120 proteins, which were homogenously monomeric, the gp140 proteins (both wild-type and disulfide-stabilized) were less homogenously trimeric (∼85% trimer) [19]; therefore, we cannot rule out the impact of the gp140-heterogeneity (with ∼15% gp140 monomer and dimer), although potentially low, on epitope-specific responses observed in the two gp140-immunized groups.

**Figure 7:**
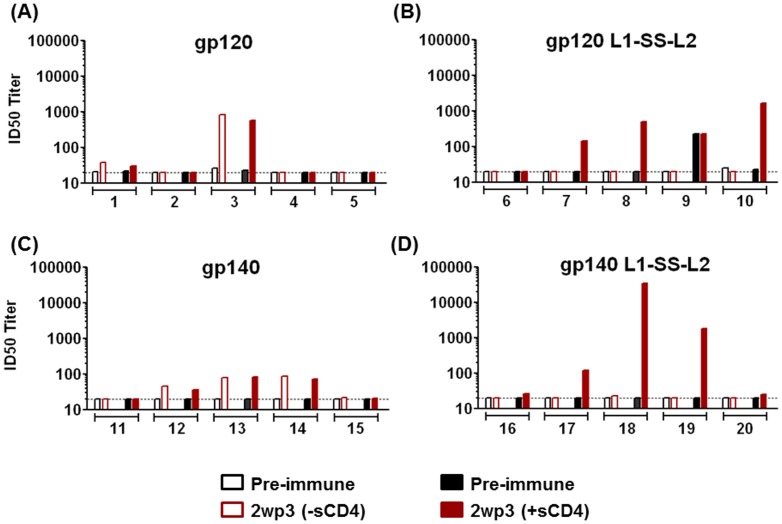
Neutralization of HIV-2 in the presence and absence of sCD4. Individual rabbit sera were tested for neutralizing activity against HIV-2 (7312A/V434M) in the absence (open bars) or presence (filled bars) of sCD4. The 50% neutralizing titers are shown for the pre-immune (black) and 2wp3 (red) serum samples. The antigens used for immunization are indicated above each graph (A – gp120, B – gp120 L1-SS-L2, C – gp140, D- gp140 L1-SS-L2); the numbers on the x-axis indicate the rabbit numbers. The dotted lines indicate the lowest dilution tested, 1∶20, that serves as the background.

The observation that both the disulfide-stabilized recombinant proteins, gp120 L1-SS-L2 and gp140 L1-SS-L2, generated significantly higher levels of binding antibodies directed to the CD4i-epitope (Figure 6A, B) led us to test if these serum antibodies would also show relatively higher neutralization properties in presence of sCD4. To do so, we used 2wp3 sera to neutralize HIV-2 (7312A/V434M), in the presence or absence of soluble CD4 (sCD4). We observed that, while none of the animals immunized by gp120 and gp140 elicited antibodies that neutralize sCD4-induced HIV-2, 3 out of 5 animals (# 7, 8 and 10) immunized with gp120 L1-SS-L2 and 3 out of 5 animals (# 17, 18 and 19) immunized with gp140 L1-SS-L2 generated antibodies that neutralized HIV-2 in the presence of sCD4 (Figure 7). These data confirm that the disulfide-stabilized immunogens used in this study elicit significantly higher levels of binding and neutralizing antibodies directed to the CD4-induced epitope.

## Discussion

Despite the promising but modest level of short-lived protection in the RV144 trial in Thailand, wherein native monomeric gp120 protein antigens were used as boost, identification and production of a superior recombinant Env-based immunogen for HIV vaccine development is warranted. Emerging results in the field continue to suggest that trimeric, functional forms of recombinant Env might generate more broadly-reactive, neutralizing antibody responses against HIV [30]–[34].

Gp120-based protein immunogens have been used in numerous pre-clinical and clinical studies. In addition to the varying glycan ‘umbrella’ protecting the underlying polypeptide epitope(s) [35] and hyper-variability in functionally less important regions [36] that all Env-based immunogens present, gp120, in particular, displays more non-neutralizing regions and undergoes larger entropic changes after receptor binding [14], [37] than trimeric/oligomeric Env. The conformational changes of the Env protein happen at both inter-domain and intra-domain levels. Not only are the alterations in the inner domain that facilitate co-receptor-engagements after CD4-binding significantly large [14], [37], the secondary structure rearrangements within the inner domain are extensive [6]. Moreover, the diversity of gp120 conformations can act as decoy to evade immune response against key neutralizing regions. Hence, restricting this inherent conformational flexibility of Env protein, via rational engineering to optimally display conserved epitopes, can potentially improve vaccine-induced immune responses while also allowing for greater stability during vaccine production. To this end, we focused on the recently described “layered” architecture in the gp120 inner domain that displays enhanced structural plasticity amongst the layers, particularly layer 2 [6]. Although we attempted several modifications (Table S1), except for stabilizing the two flexible topological layers (layers 1 and 2), the rest of the mutations failed to generate a homogenous, disulfide-stabilized gp120 that was conformationally active (i.e., bound CD4 and underwent CD4-induced conformational change to allow CD4-directed antibody, 17b, binding). In addition, the role of layer 3 in structural rearrangements were not entirely clear, until recently [38]. After rigorous *in vitro* analysis, we concluded that insertion of a single disulfide bond [in specific positions in layer 1 (V65C) and layer 2 (S115C)] was sufficient to ‘lock’ gp120 in its CD4i-state, as evident from kinetic analysis with the CD4i-site-directed mAb 17b (Figure 3), and generate conformationally well-folded Env proteins.

We confirmed the formation of the disulfide bond at the engineered site in gp120 L1-SS-L2 using high-resolution LC/ESI-FTICR mass spectrometry that allowed detection of the disulfide bond between 65C and 115C (Figures S2 and S4). During the analysis, we observed variability in disulfide bonding, particularly in the C1-V1/V2 region of the protein. This variability is consistent with recent reports that have directly [39] or indirectly [40] highlighted that alternative connection of disulfide linkages is possible in a number of recombinant envelope glycoproteins generated from CHO and HEK 293T cells. Concomitant comparative antibody binding analysis using SPR of wild-type gp120 and disulfide-stabilized gp120 (gp120 L1-SS-L2) also confirmed the improved exposure of the CD4i-site on the disulfide-stabilized gp120. Due to technical challenges, we limited both the analytical analysis for disulfide-bond configuration (via mass spectrometry) and affinity of mAb binding (via SPR) to gp120 s.

In the past, we and others have attempted to stabilize the gp120 conformation [16], [28], [41], [42]. While sCD4 or CD4-mimetic compounds stabilized gp120 and exposed the CD4i-site, these molecules blocked the key conserved receptor-binding epitope and inhibited the generation of antibody responses against the CD4BS region [28], [41], [42]. Dey et al. performed stabilization of gp120 via combination of cavity filling mutations and inter-domain (between inner and outer domain) 2-4 disulfide linkages on a core-gp120 protein with partial or large V3 and/or V1V2 loop deletions [16]. A direct comparison of effects observed *in vitro* with disulfide-stabilized, full-length gp120/gp140 described here to that of Dey et al. [16] is not feasible since the latter contained only elements of gp120 core. Regardless, the stabilized gp120 core immunogens generated antibody responses predominantly directed to the CD4i-site. In this report, although we observed a similar effect of improved antibody responses to the CD4i-site, due to the presence of the variable loop regions (V3, V1V2), we also could observe the modulation of responses to those epitopes, both in disulfide-stabilized gp120 and gp140. When comparing the immunogenicity of all four immunogens (gp120, gp120 L1-SS-L2, gp140, gp140 L1-SS-L2), the reactivity to the CD4BS across all groups was found to be similar; however, for the disulfide-stabilized gp120 and gp140, the reactivities to the CD4i-site, were higher (Figure 6 and 7). Both these *in vivo* responses are predictive of the *in vitro* binding analysis studied for CD4BS and CD4i-site. However, although V3-reactivity was lower in all groups in comparison to the group immunized with gp120, the V1V2-reactivity was very different between wild-type (gp120/gp140) and disulfide-stabilized (gp120 L1-SS-L2/gp140 L1-SS-L2) Env proteins: higher V1V2-reactivity in wild-type gp120 than its disulfide-stabilized counterpart, but lower V1V2-reactivity, although non-significant, in wild-type gp140 than the disulfide-stabilized gp140 (Figure 6). Although it is difficult to elucidate the mechanistic details of this observed response, we can speculate about the differences. It is plausible that the inserted disulfide linkage between layer 1 and 2, which is at the base of the V1V2 loop [6], restricts the mobility and presentation of V1V2 loop in gp120. However, how this restriction is then surmounted in gp140 is yet unclear.

Although the analysis using mutant forms of gp120 s to dissect epitope-directed binding antibody responses in the vaccine sera is useful as observed here, it is simplified by analyzing a specific set of epitopes only. In addition, there is a possibility of overlaps amongst epitopes in the mutant forms that could contribute to some of the observed differences amongst the wild-type and the disulfide-stabilized recombinant Env. Therefore, combination of approaches [27], [43]–[46] are often necessary for improved dissection of antibody-specificity in vaccine or HIV-infected sera.

When analyzing the epitope-directed response generated by all four Env immunogens, we observed that both the disulfide-stabilized gp120 and gp140 generated significantly higher levels of antibodies directed to the CD4i-site (Figures 6 and 7). As opposed to past CD4/mini-CD4/CD4-mimetic based liganded approaches [28], [41], [42] that faced a limitation in generating antibodies directed to the CD4BS (since the ‘pocket’ was occupied), our ‘single disulfide-linked’ approach circumvented this limitation and elicited CD4BS-directed binding antibodies (Figure 6). Since diverse relationships exist among epitope specificity, neutralization and effector function [47], generating broader ‘protective’ antibody responses to the CD4BS, V1V2, C1 and C5, and CD4i epitopes will contribute to improving efficacy of vaccine(s) against HIV. In addition, as opposed to the restrictive function of CD4i-directed antibodies suggested earlier [48], several recent studies have emphasized the role of antibodies targeting the CD4i epitope [41], [49] and demonstrated the diversity in specificity and functionality of these antibodies [50].

Overall, although conformational flexibility and structural plasticity exists between unliganded and liganded forms of HIV-1 Envs, the extent varies between Envs and the ligand inducing the conformational changes [5]. Regardless, it is without doubt that the diversity of conformations displayed by HIV-1 Env allow for an effective decoy to occlude immune responses against conserved functional regions. Hence, rational efforts such as this that limit conformational diversity to enhance immune responses to conserved epitopes in HIV-1 Env are a step forward towards the generation of improved vaccine immunogens.

## Materials and Methods

### Ethics Statement

The study was fully approved (approval no. 09 NVD 044.3.3.09) by the Institutional Animal Care and Use Committee at Novartis in accordance with the requirements for the humane care and use of animals as set forth in the Animal Welfare Act, the Institute for Laboratory Animal Research (ILAR) Guide for the care and use of laboratory animals, and all applicable local, state and federal laws and regulations.

### Reagents

Monoclonal antibody (mAb) b12 was purchased from Polymun Scientific (Vienna, Austria). Soluble CD4 (sCD4) was purchased from Progenics Pharmaceuticals (Tarrytown, NY). The mAb 17b was provided by Dr. James Robinson. MAb VRC01 was generated in-house by cloning the sequences of VL and VH chains (Protein Data Bank [PDB]: 3NGB) [51] into proprietary Ab-expression vector backbone containing the human CL and CH chains. The pDNA was then used to transfect HEK 293 cells and the expressed mAbs-containing supernatants were dia-filtered and concentrated 10× before purifying using Protein A-affinity chromatography. The gp120 mutants were generated and validated, as described previously [27], [28].

### Generation of recombinant proteins

Recombinant HIV-1 envelope (Env) glycoproteins, gp120 and gp140, were derived from the subtype B CCR5-tropic strain HIV-1 SF162 and were produced by transfection of HEK 293 cells. The layers 1 and 2 disulfide-stabilized gp120 (gp120 L1-SS-L2) and gp140 (gp140 L1-SS-L2) were also derived from SF162 and produced in HEK 293 cells. All four glycoproteins were purified using a three-step purification process involving Galanthus Nivalis-Agarose (GNA) affinity column, cation-exchange DEAE column and a final ceramic hydroxyapatite (CHAP) column as described by Srivastava et al. [52]. Purified glycoproteins were then analyzed by SDS-PAGE (for level of purity) and immunoblots for specific reactivity (to anti-SF162 gp140 polyclonal rabbit sera). The purified glycoproteins were homogeneous (>95% monomer for gp120 s; >80% trimer for gp140 s) with purity of >98%. Endotoxin levels in glycoproteins were measured using Endosafe® cartridges and an Endosafe®-PTSTM spectrophotometer (Charles River Laboratories International, Inc., Wilmington, MA), and found to be ≤0.05 EU/immunization dose.

Other variants of SF162 gp120 used for epitope-mapping purposes, such as gp120ΔV3, gp120ΔV1V2, gp120 D368R (CD4-binding site mutant), and gp120 I420R (CD4i site mutant), were also produced and purified, as described above and previously [28]. Gp140-miniCD4 complex was generated by covalently linking a CD4-mimetic miniprotein, miniCD4 (M64U1-SH), to trimeric gp140 using site-specific disulfide linkages as described previously [28].

Carbopol® 971P NF (referred to as Carbopol971P in this study) was purchased from Lubrizol as powder and was then resuspended in water under sterile conditions to generate a 0.5% homogenous, low viscosity suspension. The suspension was stored at 4°C until further use. A 1∶1 (v/v) mix of gp140 protein and 0.5% (w/v) Carbopol971P (pH≥3.0) was made for all in vitro evaluations. For administration in animals, a 1∶1 (v/v) mix of gp140 and 0.5% (w/v) Carbopol971P was first made and the gp140 protein-Carbopol971P complex incubated for 30 minutes before addition of an equal volume of MF59, thereby keeping the final concentration of Carbopol971P suspension administered at 0.125% (w/v). For all Carbopol971P suspensions for in vivo studies, endotoxin levels were measured using Endosafe® cartridges and an Endosafe®-PTSTM spectrophotometer (Charles River Laboratories International, Inc., Wilmington, MA).

### Analysis of binding affinity using Surface Plasmon Resonance

To determine the binding affinities of wild-type gp120 and the disulfide-stabilized gp120, gp120 L1-SS-L2, we used Surface Plasmon Resonance (SPR)-based BIAcore 3000. ∼200 RU of sCD4 or mAbs b12, VRC01 or 17b were immobilized directly onto a CM5 sensor chip via amine coupling. Varying concentrations of gp120 s, either wild-type of disulfide-stabilized, were then injected at 80 µl/min. In the case of +sCD4, the gp120 proteins were pre-incubated with 2-fold molar excess of sCD4 for 1 hour at room temperature before injection. The binding analyses were performed at 25°C with HBS-EP buffer as running buffer. The experimental curves were then fitted to a 1∶1 Langmuir binding model using BIAevaluation software 3.2 (BIAcore Inc). The association rate, Kon, dissociation rate, Koff, and the dissociation constant, Kd, which are derived following the 1∶1 fit, are indicated in Figure 3.

The binding of CCR5 peptide to the gp120 proteins, wild-type and disulfide stabilized, in the presence or absence of soluble CD4 (sCD4) were performed as described previously [21]. Approximately 5000 RU of 22-mer tyrosine-sulfated biotinylated N-terminal CCR5 peptide [21] was immobilized on to a SA chip and 100 nM of gp120/gp120 L1-SS-L2 proteins were injected at 10 µl/min, either alone or upon pre-incubation with sCD4 (2-fold molar excess, as described above). The binding analyses were performed at 25°C with HBS-EP buffer as running buffer.

### Analysis of disulfide bond using LC/ESI-FTICR MS

To determine disulfide bonding, particularly between the two engineered cysteines at position 65 and 115, 75 µg of SF162 gp120 L1-SS-L2 protein was alkylated with 4-vinylpyridine, deglycosylated with PNGase F and then digested with trypsin, as described previously [39]. Following tryptic digestion, samples were analyzed using a hybrid linear ion trap Fourier Transform-Ion Cyclotron Resonance (LTQ-FTICR) mass spectrometer coupled with a Waters NanoACQUITY UltraPerformance Liquid Chromatography (UPLC) system. Chromatographic separation was performed using a C18 PepMap 300™ column (150 mm×300 µm i.d. 5 µM, 300Å; LC Packings, Sunnyvale, CA) that is equilibrated with 97% mobile phase A (99.9% water with 0.1% formic acid) and 3% mobile phase B (99.9% acetonitrile with 0.1% formic acid). Approximately 5 µL of sample was injected into the column at a flow rate of 5 µL/min using the following gradient: a linear increase to 40% B in 50 minutes then to 90% B in 10 min. The column was held at 90% B for 10 min before equilibration. Data were collected in a data dependent acquisition mode in which the five most intense ions in a high resolution survey scan in the FTICR cell were sequentially and dynamically selected for subsequent collision-induced dissociation (CID) in the LTQ linear ion trap to ascertain the disulfide connectivity, as described previously [39].

### Rabbit immunizations

Immunization studies were conducted at Josman LLC (Napa, CA), a research facility that is licensed through the USDA (No. 93-R-0260) and has a Public Health Service (PHS) Assurance from the NIH (No. A3404-01). Four groups of New Zealand White rabbits (5 young adult females per group) were used in the study. Rabbits were immunized with SF162 gp140 protein adjuvanted with either MF59 alone, Carbopol971P alone, or with Carbopol971P plus MF59. Three immunizations were administered intramuscularly, in the gluteus muscle (2 sites per immunization), at weeks 0, 4, and 12. The protein dosage at each immunization was 25 µg. Serum samples were prepared from blood collected prior to the first immunization (pre-bleed) and at various time-points post each immunization (2wp1, 2wp2, 2wp3, 4wp3 and 8wp3) and analyzed for binding and neutralizing antibody responses.

For assessment of local reactogenicity associated with injection of 0.125% (w/v) Carbopol971P, visual observations of skin for edema and erythema at injection sites were performed pre-dose, immediately after immunization, and 24 h and 48 h after immunization. General observations for any obvious clinical signs were performed immediately after immunization, and 24 and 48 h post-immunization. Body-weights were also recorded before the beginning of the study, before each immunization, and 24 and 48 h following each immunization.

The study was fully approved by the Institutional Animal Care and Use Committee at Novartis (approval no. 09 NVD 044.3.3.09) in accordance with the requirements for the humane care and use of animals as set forth in the Animal Welfare Act, the ILAR Guide for the care and Use of Laboratory Animals, and all applicable local, state and federal laws and regulations.

### Envelope-specific antibody ELISA and avidity measurements

Envelope-specific total antibody titers in sera from rabbits immunized with Carbopol971P plus MF59-adjuvanted wild-type gp120, the disulfide-stabilized gp120 (L1-SS-L2 gp120), wild-type gp140 and the disulfide-stabilized gp140 (L1-SS-L2 gp140) were quantified by a standard ELISA assay using SF162 gp140 protein, as previously described [52]. The GMT ELISA titers indicated here are based on end-point dilution titers. Antibody avidity index determination was performed using an ammonium thiocyanate (NH_4_SCN) displacement ELISA as described elsewhere [52]. All samples were tested in duplicate and results were expressed as the NH_4_SCN concentration required to displace 50% of the bound serum antibodies.

### Mapping of epitope-specific antibody binding

To determine epitope-specific antibodies in 2wp3 sera from rabbits immunized with Carbopol971P plus MF59-adjuvanted wild-type gp120, disulfide-stabilized gp120 (L1-SS-L2 gp120), wild-type gp140 and the disulfide-stabilized gp140 (L1-SS-L2 gp140), binding of sera to wild-type gp120 (for gp120 based groups) and wild-type gp140 (for gp140 based groups) were used as binding references (100%-binding). The mutant (SF162) gp120 s (gp120ΔV1V2 – for V1V2-specific reactivity; gp120ΔV3 – for V3-specific reactivity; gp120D368R – CD4BS mutant, for CD4BS-reactivity; and gp120I420R – CD4i-site mutant, for CD4i-site reactivity) were generated and the ELISA performed using the D7324 Ab-capture protocol, as previously described 28,53. The bound serum antibodies were then washed and detected using a goat anti-rabbit IgG Fc antibody conjugated to horseradish peroxidase, and the optical density (OD) determined using a microplate reader (Molecular Devices) at 450 nm. Percent (%) epitope-directed binding was calculated by comparing the binding of sera to wild-type gp120 and mutant gp120 s. Thus, % epitope-directed binding  =  (EC50 of binding to gp120 ΔV3/ΔV1V2/D368R/I420R÷EC50 of binding to gp120 WT) ×100.

### HIV-1 neutralization assays

Virus neutralization titers were measured using a well-standardized assay employing pseudoviruses and a luciferase reporter gene assay in TZM-bl cells [Dr. John C. Kappes, Dr. Xiaoyun Wu and Tranzyme, Inc. (Durham, NC)] as described previously [46], [47]. Briefly, 200 TCID50 pseudoviruses/well were added to diluted serum samples and incubated at 37°C for 1 h. Following incubation, 10,000 cells/well in DEAE-dextran-containing media were added and incubated for 48 h at 37°C. The final concentration of DEAE-dextran was 10 µg/ml. After a 48 h incubation, 100 µl of cells were transferred to 96-well black solid plates (Costar) for measurements of luminescence using Bright-Glo substrate solution as described by the supplier (Promega). Neutralization titers are the dilution at which relative luminescence units (RLU) were reduced by 50% compared to virus control wells after subtraction of background RLUs. HIV-1 Env pseudoviruses were prepared by co-transfection of 293T cells with expression plasmids containing full-length molecularly cloned gp160 env genes from a panel of HIV-1 isolates combined with an env-deficient HIV-1 backbone vector (pSG3Δenv) using FuGENE-6 HD (Roche Applied Sciences, Indianapolis, IN), as previously reported [47]. After 48 h, the cell culture supernatants containing the pseudoviruses were filtered through a 0.45 µm filter and stored at −80°C until use.

### HIV-2 neutralization assays

For the detection of CD4i neutralizing antibodies, a modified neutralization was used, as previously described [49].

### Statistical analyses

Comparisons between multiple groups were carried out using analysis of variance (1 way ANOVA). A Kruskal-Wallis test was used to analyze differences between multiple epitope-directed groups. For all comparisons, a two-sided P<0.05 was considered statistically significant. All analyses were performed using the analysis software within the GraphPad Prism package 5.01.

## Supporting Information

Figure S1 **Primary sequence alignment and schematic representation of proximity of layer 1 and layer 2 and insertion of a specific cysteine pair in gp120 inner domain.** (A) Primary sequence alignment of SF162 gp120 and HxB2 gp120, highlighting the two point mutations in layer 1 (V65C) and layer 2 (S115C) regions of the gp120 sequence. (B) Ribbon diagram representation showing the targeted position in layer 1 (magenta, Val65) and layer 2 (green, Ser115), which are proximal and separated by 3.7Å, for generating the disulfide bond in the gp120 inner domain. Layer 1 and Layer 2, here on are referred to as L1 and L2, respectively.
(TIF)

Figure S2 **Summary of the disulfide linkages in SF162 gp120 L1-SS-L2 as detected by LC/ESI-FTICR MS.** (A) Major forms of disulfide-linked tryptic peptides, with * highlighting the formation of a disulfide bond via the engineered cysteine residues at position 65 and 115. (B) Minor forms of disulfide-linked tryptic peptides.
(TIF)

Figure S3 **Surface Plasmon Resonance (SPR) binding analysis to show qualitative binding of gp120 proteins, wild-type and disulfide stabilized, in presence or absence of soluble CD4 to immobilized CCR5 peptide.** Disulfide-stabilized SF162 gp120 (gp120 L1-SS-L2; orange) bound to the biotinylated N-terminal CCR5 peptide [21], immobilized on SA chip, ∼5-fold better (at time  = 300 s) than the wild-type gp120 (cyan). When pre-incubated with soluble CD4 (sCD4), both complexes, gp120+sCD4 (blue) and gp120 L1-SS-L2+sCD4 (red), bound CCR5 peptide with increased affinity relative to their unbound gp120 counterparts, as expected (gp120+sCD4 bound >10-fold better than gp120, gp120 L1-SS-L2+sCD4 bound ∼4-fold better than gp120 L1-SS-L2). In addition, gp120 L1-SS-L2+sCD4 complex exhibited ∼2-fold increased binding to CCR5 as compared to gp120+sCD4 binding to CCR5. The black dotted curve shows binding of buffer (HBS-EP) alone, as a control, to the immobilized surface.
(TIF)

Figure S4 **Spectra of the new site-specific disulfide in SF162 gp120 L1-SS-L2.** (A) Collision-induced dissociation (CID) and (B) Electron-transfer dissociation (ETD) spectra of gp120 L1-SS-L2 to validate that the new disulfide mass is correct and verify the disulfide connectivity shown in Figure S3. (C) Mapping the fragment ions from the (CID and ETD) spectra to the gp120 sequence to highlight the formation of the new disulfide and represent the same in the schematic on right.
(TIF)

Table S1 **List of gp120 mutants with their site-specific (position numbering based on HxB2) cysteine mutations and their** ***in vitro*** **analysis leading to immunogenicity testing.** List of cysteine mutations in layer 1 (L1), layer 2 (L2) and layer 3 (L3) of gp120 (to allow disulfide, S-S, bond formations) as well as a summary of expression, purification and *in vitro* CD4/CD4i-directed antibody (17b) binding (via SPR) of those gp120 mutants to justify their advancement to the rabbit immunogenicity study.
(TIF)
