# Supplementary figures and images for: Stabilizing Exposure of Conserved Epitopes by Structure Guided Insertion of Disulfide Bond in HIV-1 Envelope Glycoprotein
Source: PLoS One. 2013 Oct 16;8(10):e76139. doi: 10.1371/journal.pone.0076139 (PMC3797752; doi:10.1371/journal.pone.0076139)

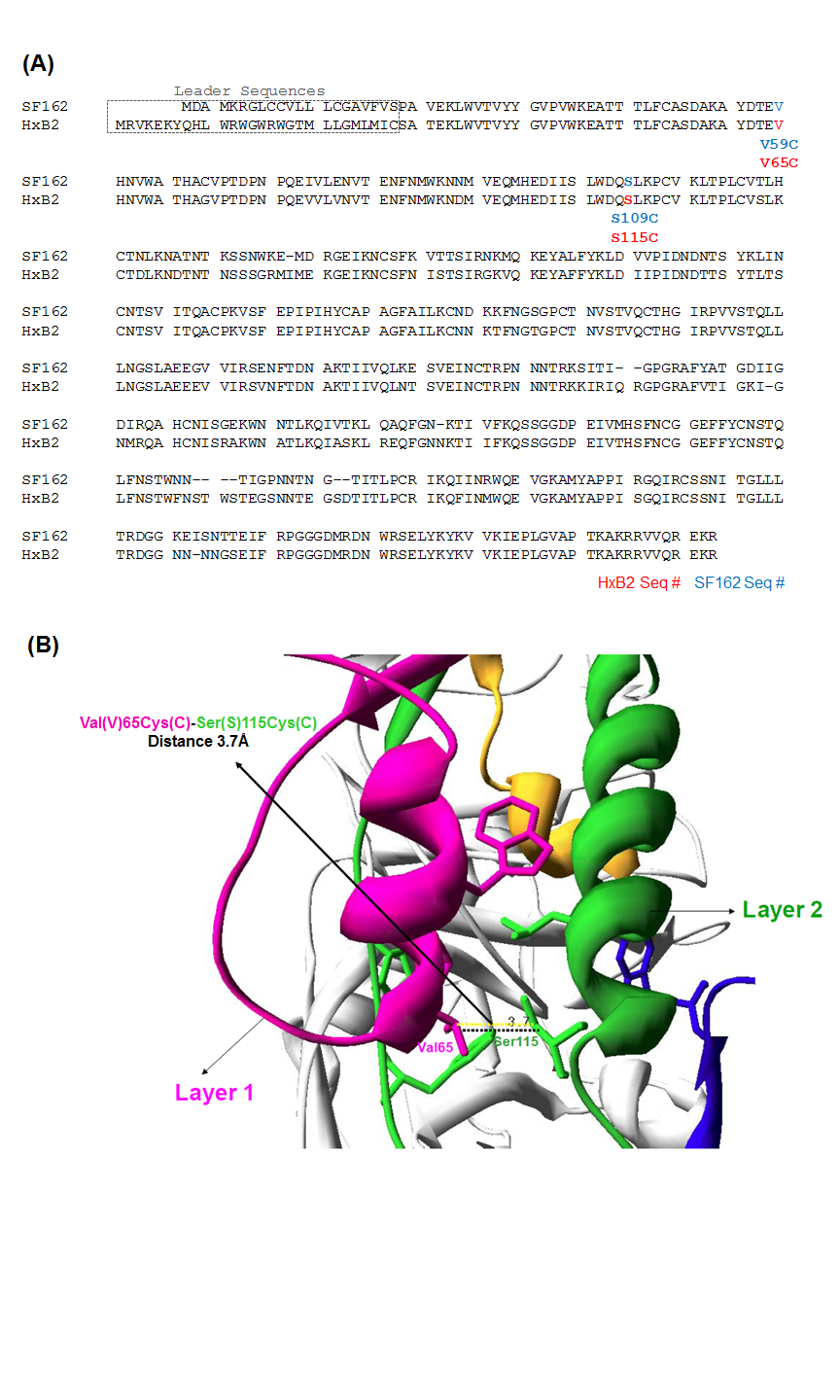

Supplement: Figure S1 — Primary sequence alignment and schematic representation of proximity of layer 1 and layer 2 and insertion of a specific cysteine pair in gp120 inner domain. (A) Primary sequence alignment of SF162 gp120 and HxB2 gp120, highlighting the two point mutations in layer 1 (V65C) and layer 2 (S115C) regions of the gp120 sequence. (B) Ribbon diagram representation showing the targeted position in layer 1 (magenta, Val65) and layer 2 (green, Ser115), which are proximal and separated by 3.7Å, for generating the disulfide bond in the gp120 inner domain. Layer 1 and Layer 2, here on are referred to as L1 and L2, respectively. (TIF) [file pone.0076139.s001.tif]

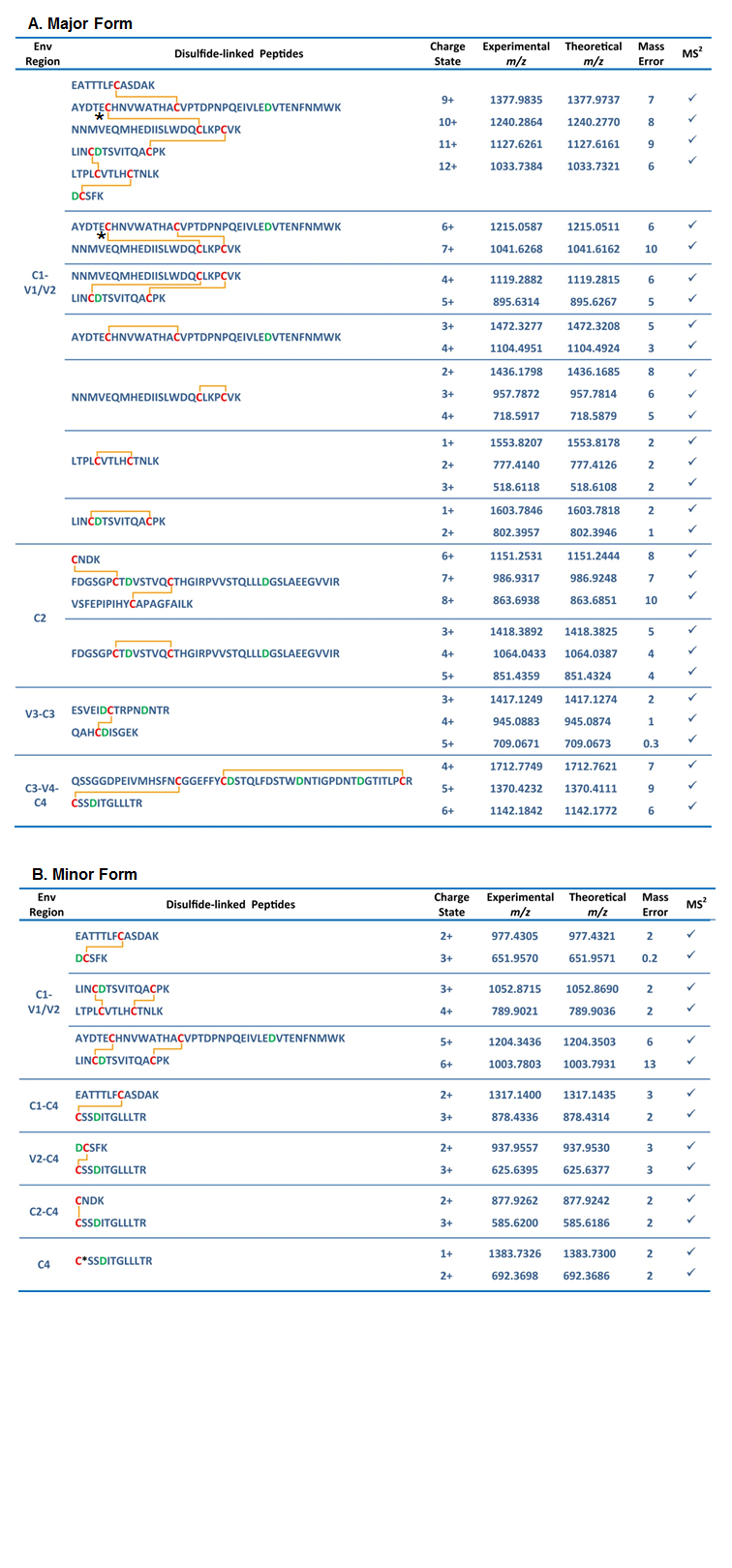

Supplement: Figure S2 — Summary of the disulfide linkages in SF162 gp120 L1-SS-L2 as detected by LC/ESI-FTICR MS. (A) Major forms of disulfide-linked tryptic peptides, with * highlighting the formation of a disulfide bond via the engineered cysteine residues at position 65 and 115. (B) Minor forms of disulfide-linked tryptic peptides. (TIF) [file pone.0076139.s002.tif]

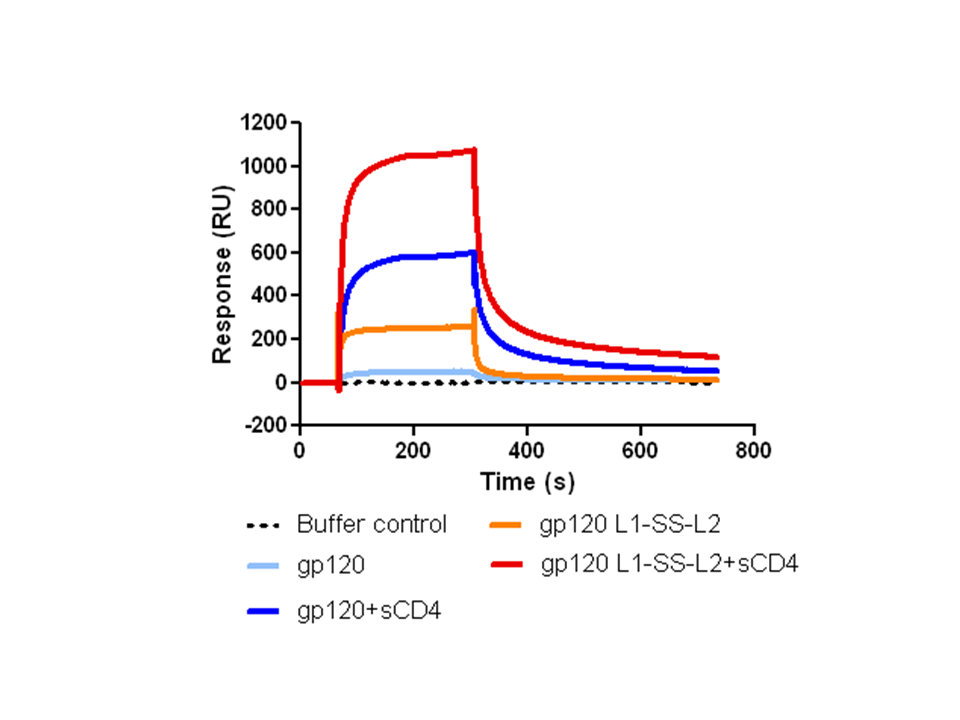

Supplement: Figure S3 — Surface Plasmon Resonance (SPR) binding analysis to show qualitative binding of gp120 proteins, wild-type and disulfide stabilized, in presence or absence of soluble CD4 to immobilized CCR5 peptide. Disulfide-stabilized SF162 gp120 (gp120 L1-SS-L2; orange) bound to the biotinylated N-terminal CCR5 peptide [21], immobilized on SA chip, ∼5-fold better (at time = 300 s) than the wild-type gp120 (cyan). When pre-incubated with soluble CD4 (sCD4), both complexes, gp120+sCD4 (blue) and gp120 L1-SS-L2+sCD4 (red), bound CCR5 peptide with increased affinity relative to their unbound gp120 counterparts, as expected (gp120+sCD4 bound >10-fold better than gp120, gp120 L1-SS-L2+sCD4 bound ∼4-fold better than gp120 L1-SS-L2). In addition, gp120 L1-SS-L2+sCD4 complex exhibited ∼2-fold increased binding to CCR5 as compared to gp120+sCD4 binding to CCR5. The black dotted curve shows binding of buffer (HBS-EP) alone, as a control, to the immobilized surface. (TIF) [file pone.0076139.s003.tif]

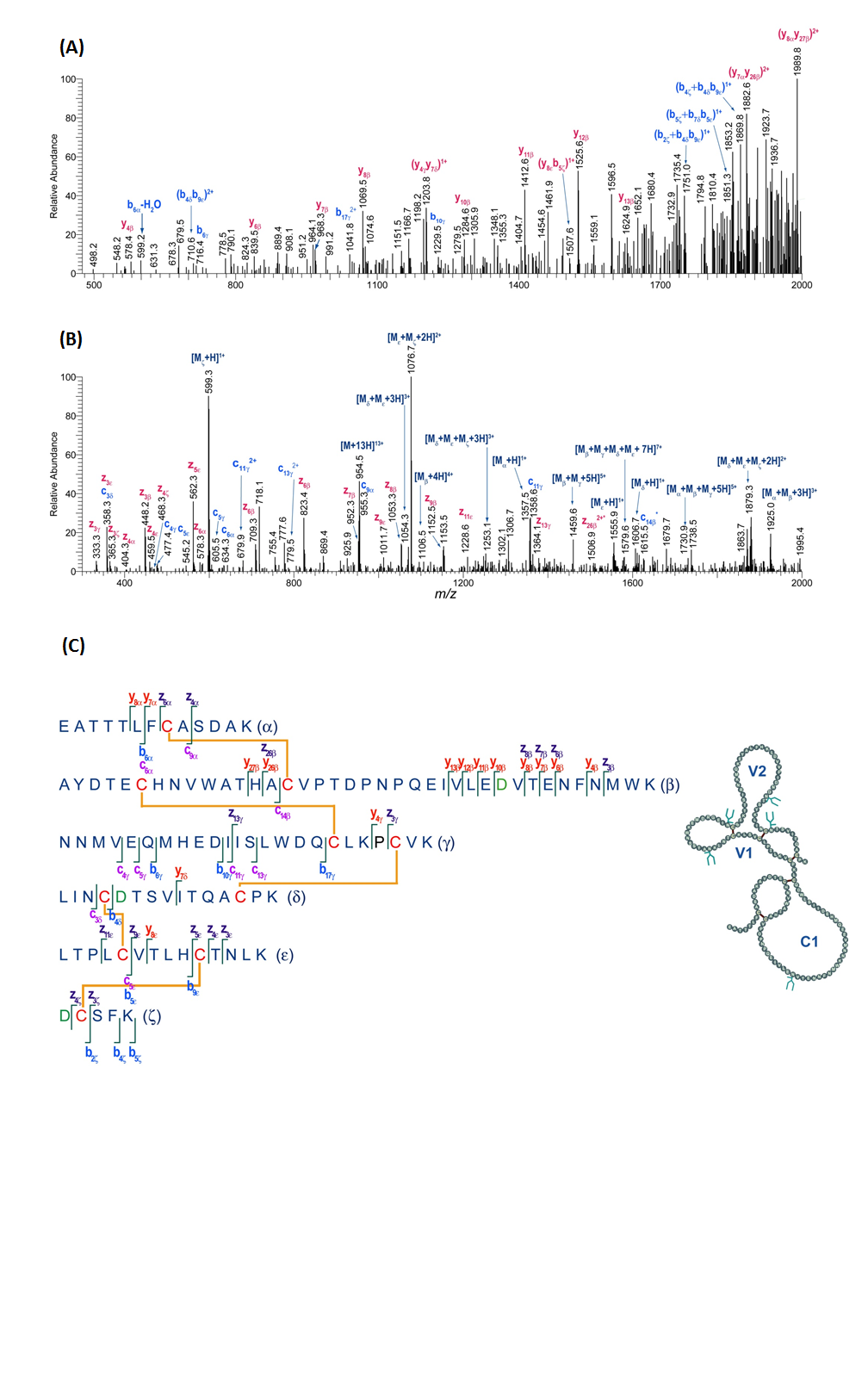

Supplement: Figure S4 — Spectra of the new site-specific disulfide in SF162 gp120 L1-SS-L2. (A) Collision-induced dissociation (CID) and (B) Electron-transfer dissociation (ETD) spectra of gp120 L1-SS-L2 to validate that the new disulfide mass is correct and verify the disulfide connectivity shown in Figure S3. (C) Mapping the fragment ions from the (CID and ETD) spectra to the gp120 sequence to highlight the formation of the new disulfide and represent the same in the schematic on right. (TIF) [file pone.0076139.s004.tif]
